# Simultaneous Kissing Balloon Inflation of the Transcatheter Aortic Valve Replacement Valve and an Ostial Coronary Stent—A Novel Coronary Protection Technique

**DOI:** 10.1016/j.shj.2022.100074

**Published:** 2022-08-17

**Authors:** Imran Baig, Arthur J. Lee, William Brinkman, Ambarish Gopal, Lakshmi Prasad Dasi, Karim Al-Azizi

**Affiliations:** a Baylor Scott & White Health The Heart Hospital - Plano, Plano, Texas, USA; b Department of Biomedical Engineering, Georgia Institute of Technology and Emory University, Atlanta, Georgia, USA

**Keywords:** Kissing balloon inflation, PCI, TAVR

## Case Report

**Figure 1:**
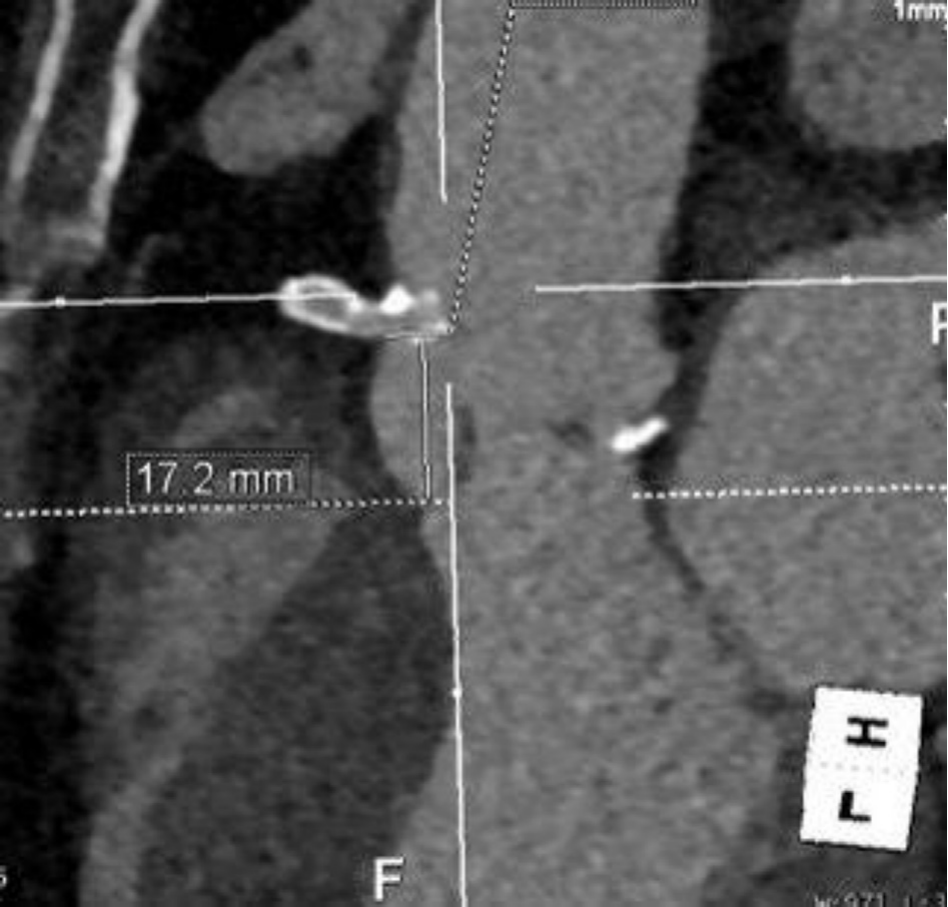
**The right coronary artery stent protruding into the sinus of Valsalva**.

**Figure 2:**
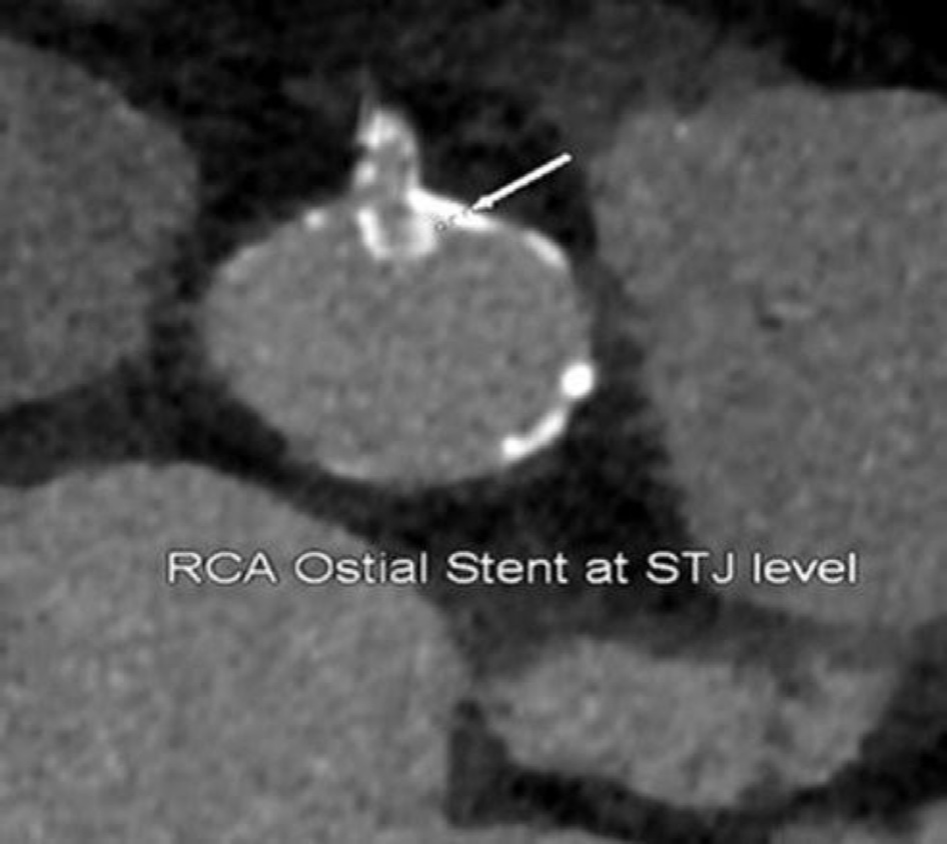
**Right coronary artery (RCA) stent protrusion into the aorta at the sinotubular junction (STJ) level**.

An 83-year-old female was seen in the valve clinic for severe symptomatic aortic stenosis. The patient underwent coronary angiography and subsequent transcatheter aortic valve replacement (TAVR) computed tomography (CT) scan. This demonstrated the previously placed proximal right coronary artery (RCA) stent to be protruding substantially into the sinus of Valsalva with inability to selectively engage the stent. CT scan demonstrated the length of protrusion to be 7 mm (Figures 1 and 2).

**Figure 3:**
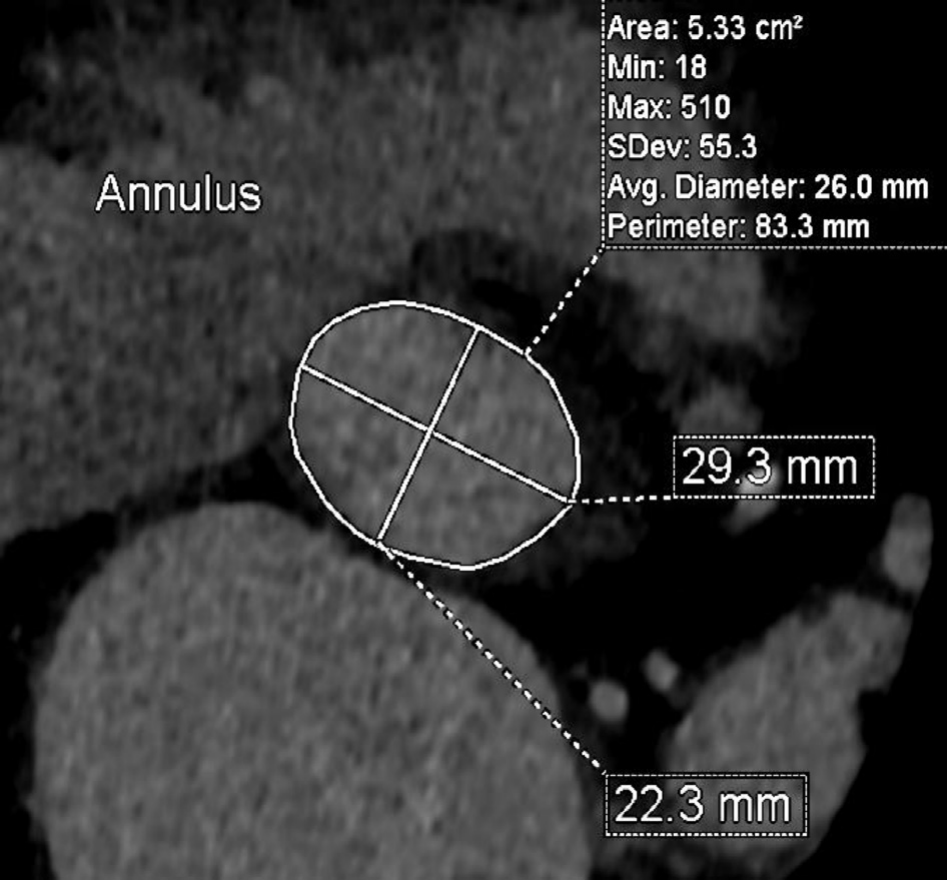
**Aortic annulus measurements**.

**Figure 4:**
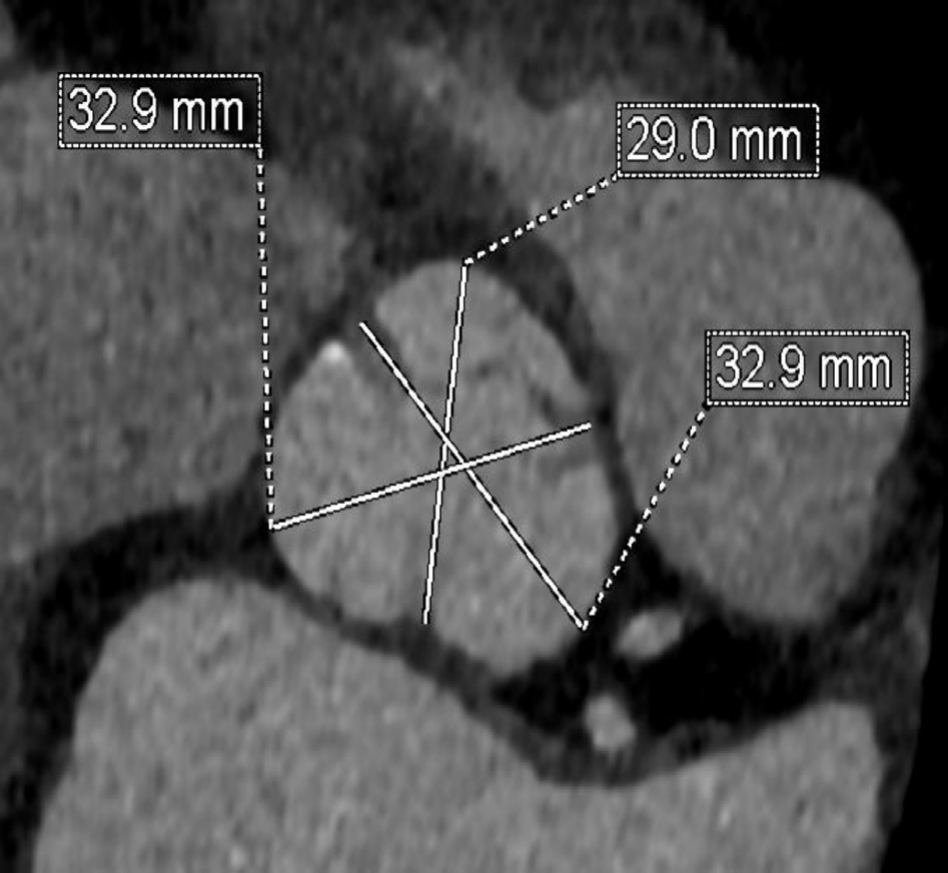
**Sinus of Valsalva measurements**.

**Figure 5:**
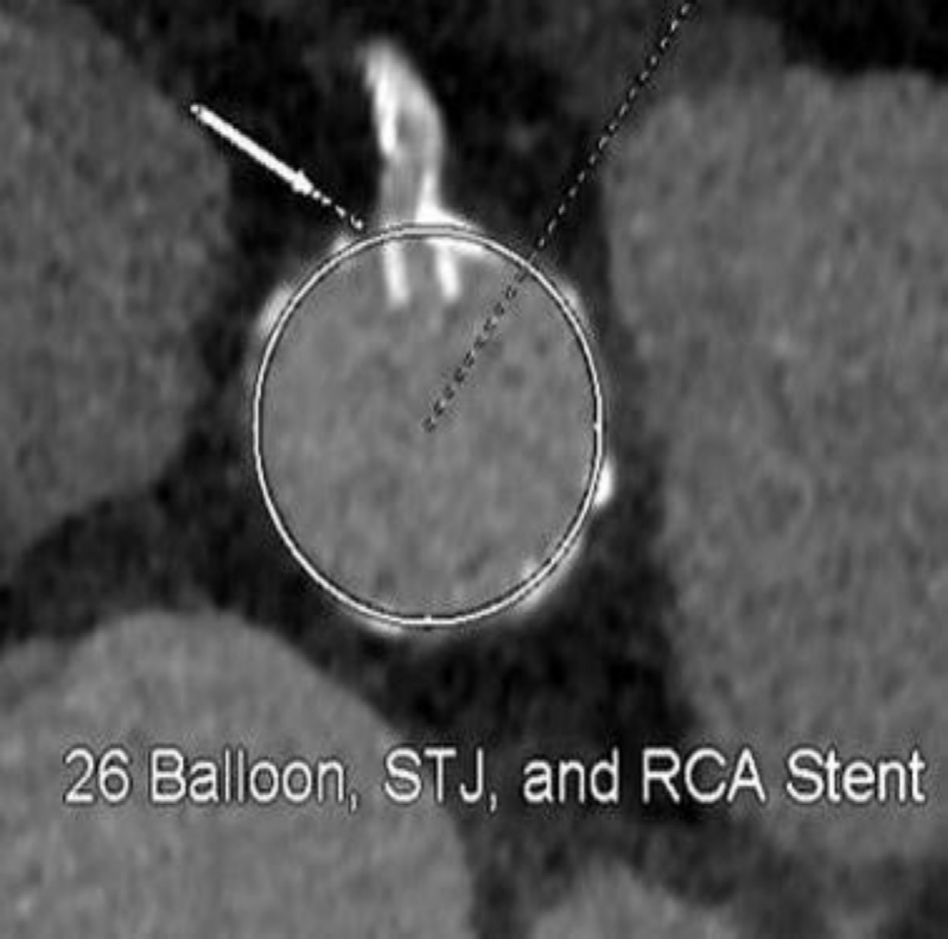
**Potential distortion of the stent with a transcatheter aortic valve replacement balloon**. Abbreviations: RCA, right coronary artery; STJ, sinotubular junction.

Preoperative CT angiography demonstrated appropriate sizing of the iliac and femoral system for transfemoral access. The aortic annulus measured 5.33 cm^2^ with a perimeter of 83.3 mm (Figure 3). The sinus of Valsalva measurements was 32 mm × 29 mm × 32 mm (Figure 4). The RCA coronary height was 17.2 mm. The team decided to proceed with a 26-mm sized Sapien 3 (S3) valve (Edwards LifeSciences, Irvine, California). There was concern regarding RCA stent distortion and possible compromise of flow to the RCA with the S3 TAVR balloon during deployment (Figure 5). DASI, an acronym for direct analytical surgical individualization, is a Health Insurance Portability and Accountability Act-compliant, cloud-based platform. It utilizes the patient’s CT scan to model the patient’s unique anatomy and then produces simulations of the procedure. These patient-specific simulations give surgeons concrete data to know what valve and what approach to use to cut down on potential complications.^1^ We utilized this advanced predictive model and reconstructions confirming the likely interaction between the valve deployment balloon and the right coronary stent by DASI simulations (Figures 11 and 12).

**Figure 6:**
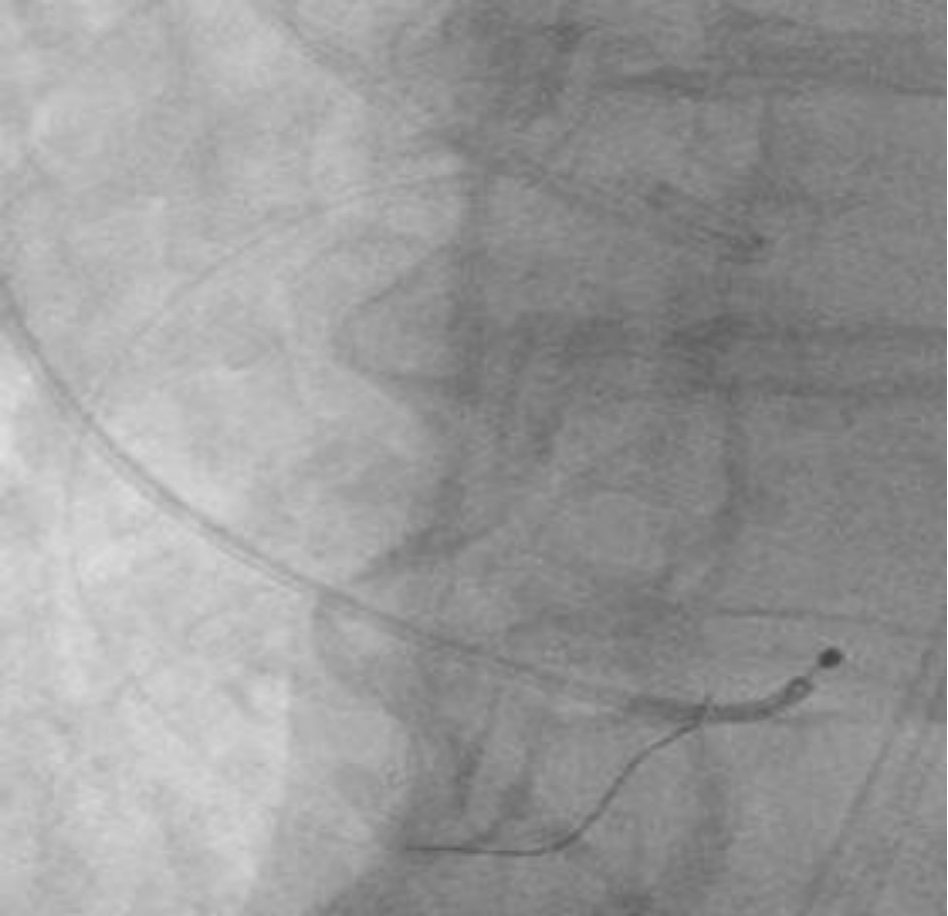
**A JR-4 guide pushed down on the cusp to facilitate wiring the true ostium of the stent**.

**Figure 7:**
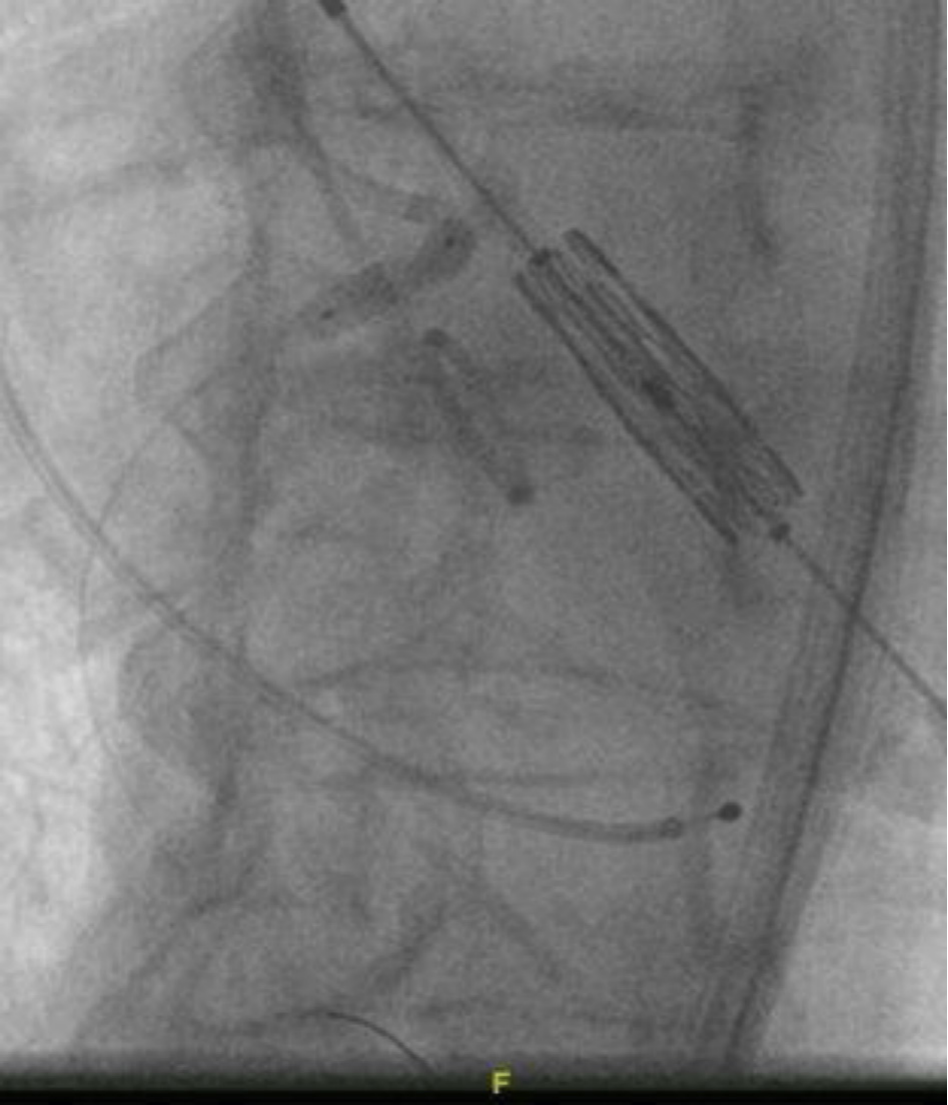
**Initial inflation of the coronary balloon to protect the pre-existing coronary stent**.

**Figure 8:**
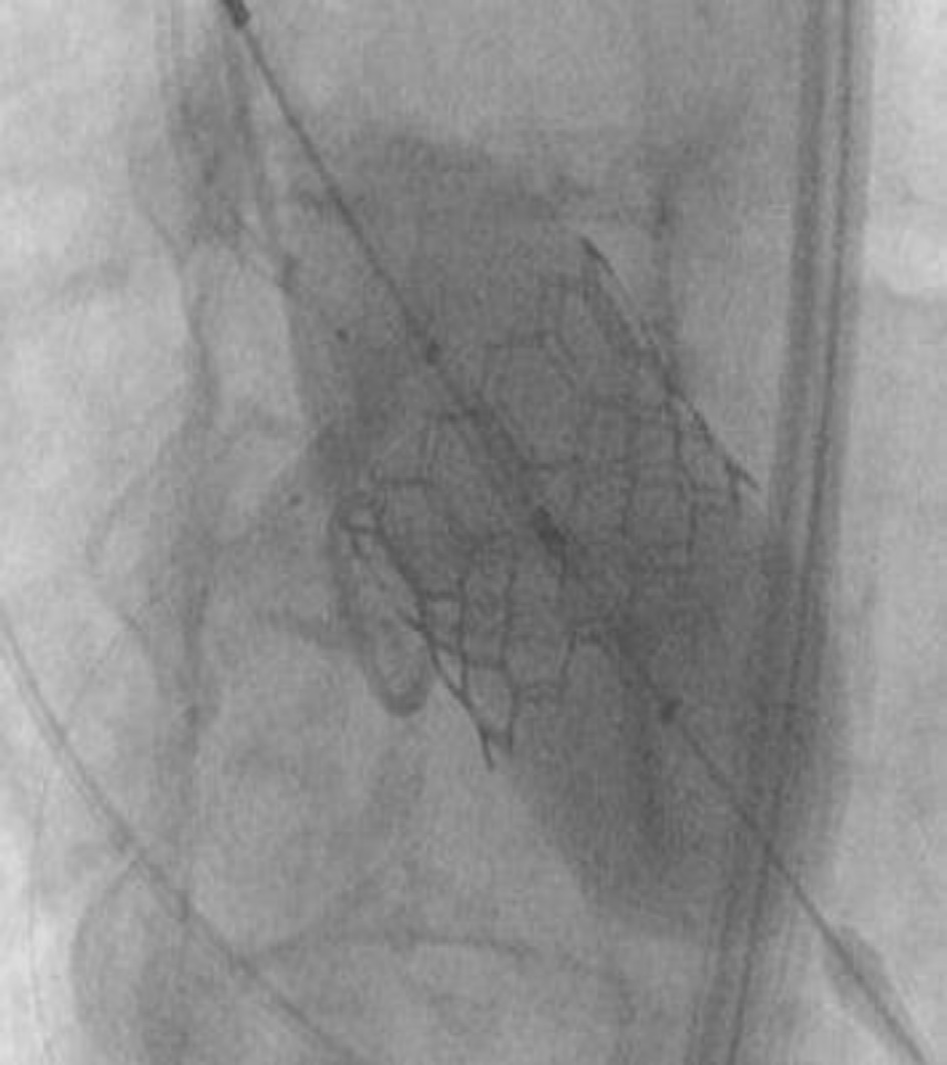
**After inflation of the coronary balloon, the transcatheter aortic valve replacement stent/balloon was deployed**.

**Figure 9:**
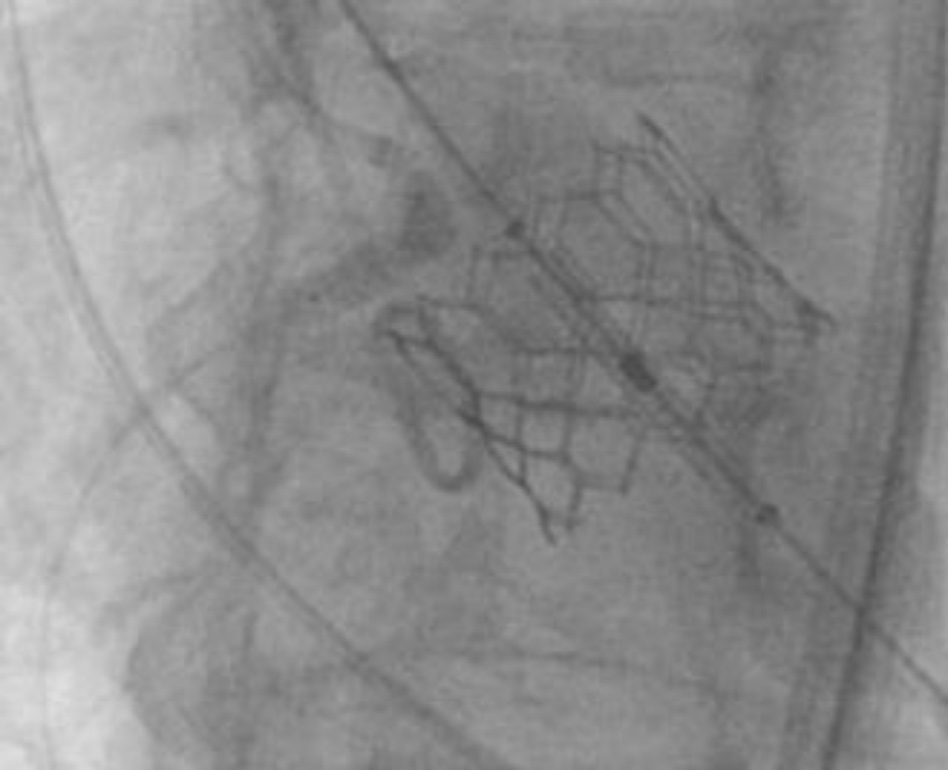
**To protect the coronary stent, the transcatheter aortic valve replacement balloon was completely deflated prior to deflating the coronary stent**.

**Figure 10:**
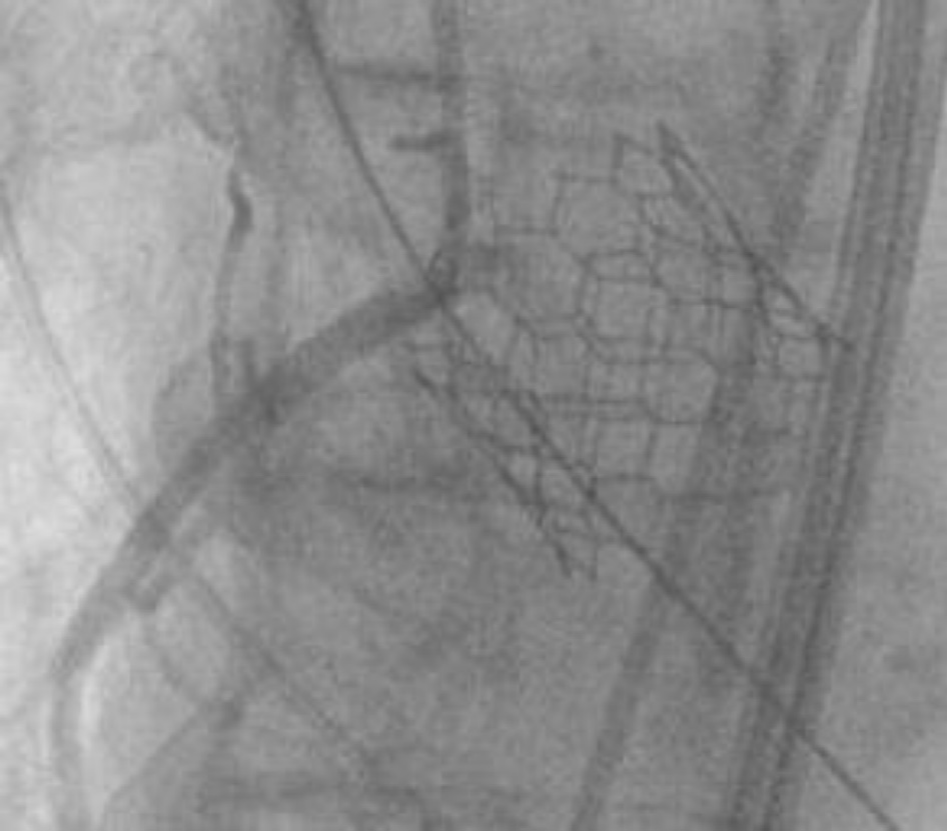
**Final angiogram demonstrated adequate protection with TIMI 3 flow and no evidence of stent distortion**. Abbreviation: TIMI, The Thrombolysis in Myocardial Infarction score

**Figure 11:**
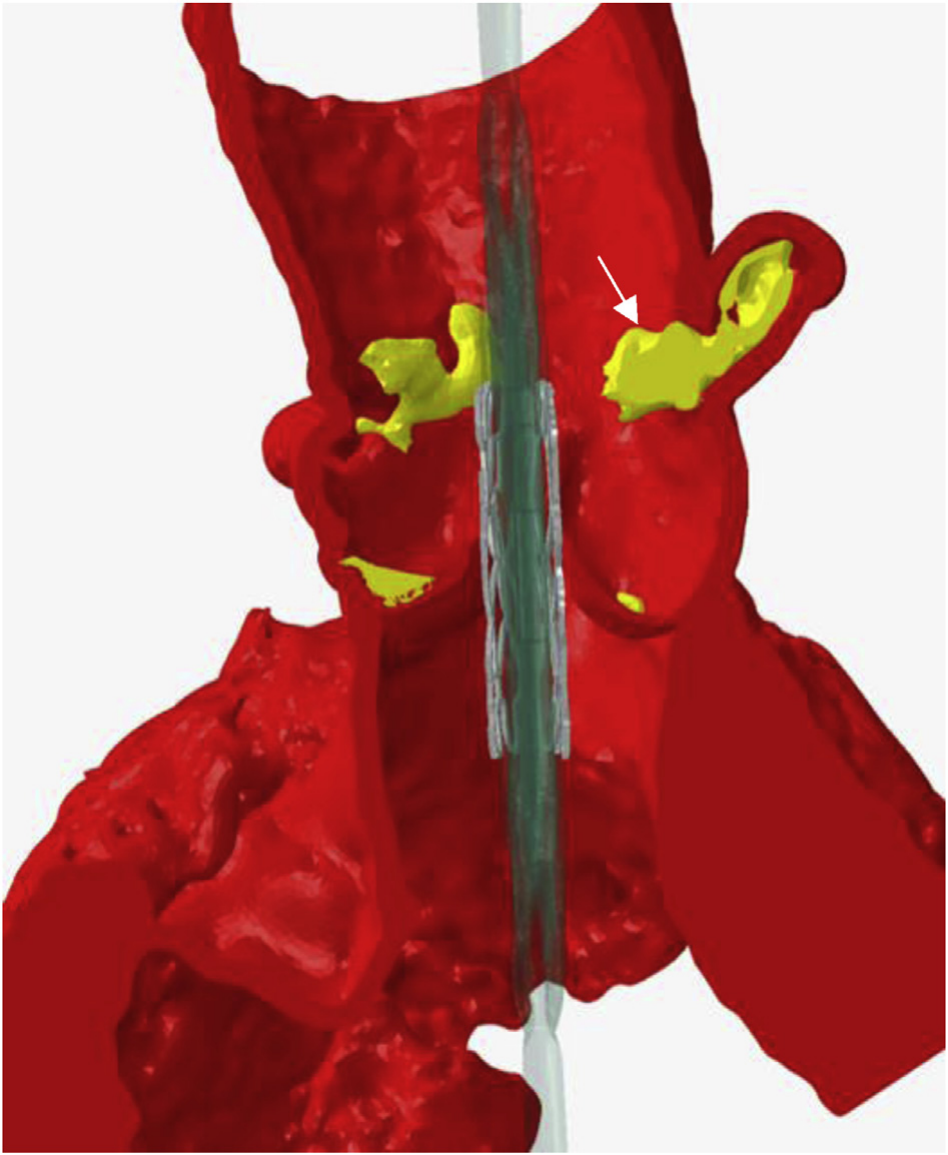
**Direct analytical surgical individualization simulation, demonstratingthe protruding ostial right coronary artery stent and its relation to the stent/balloon (arrow)**.

**Figure 12:**
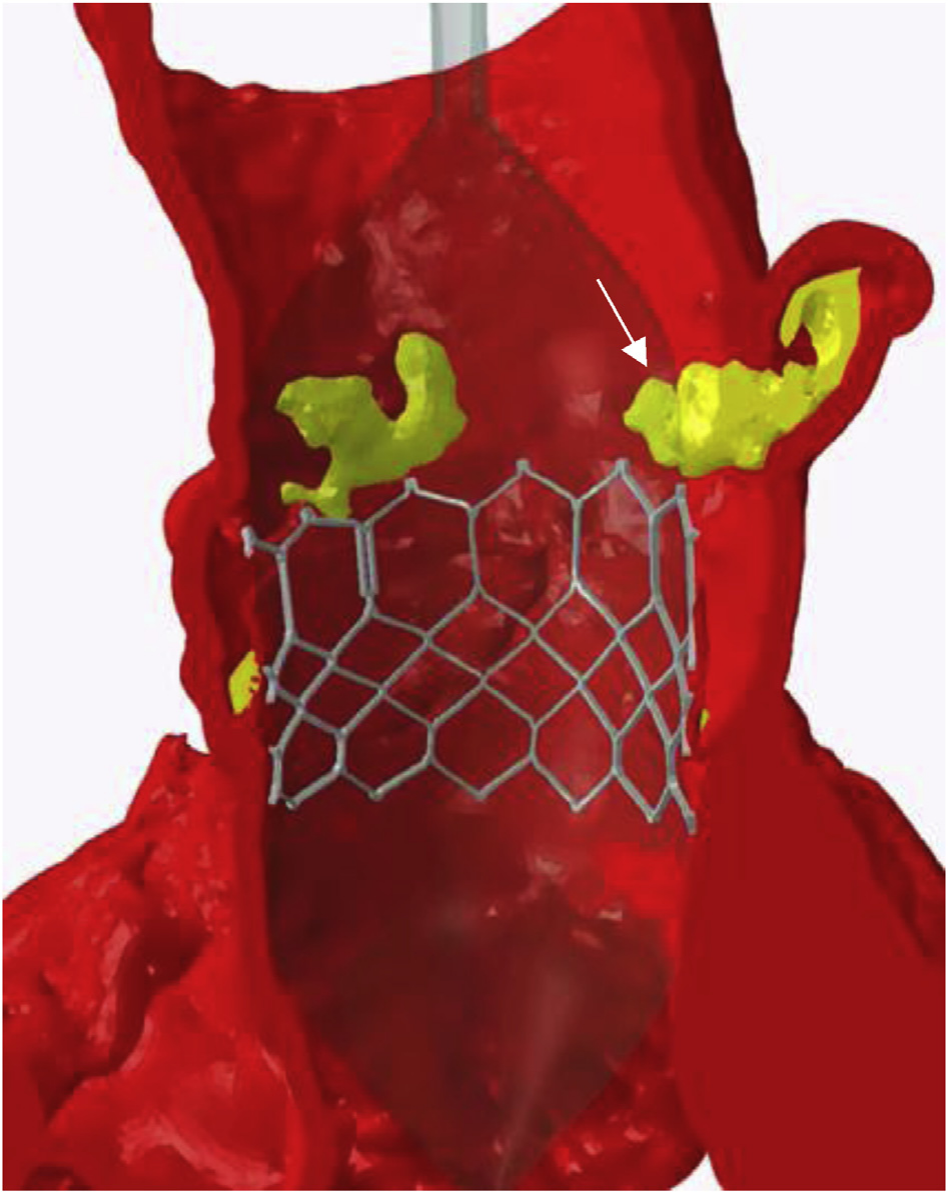
**Direct analytical surgical individualization simulation,** demonstrating **deformation of the right coronary artery stent (arrow) during transcatheter aortic valve replacement deployment**.

The heart team’s decision was to move forward with protecting the RCA during deployment. A JR-4 guide was maneuvered down into the aortic cusp, and a 0.014-mm coronary wire was free wired carefully into the RCA. This was done to ensure entry through the true orifice instead of a side stent strut (Figure 6). A 4.0 noncompliant balloon was placed in the RCA. The TAVR valve was than inserted through the right common femoral sheath and placed across the aortic valve in the usual fashion. The right ventricle was paced at 180 beats per minute. The RCA balloon was then inflated at 12 atmospheres with half the balloon protruding into the sinus (Figure 7). We then deployed the 26 S3 TAVR valve (Figure 8). After complete deflation of the TAVR stent, the RCA balloon was deflated (Figure 9). This critical step enabled us to prevent any distortion of the coronary stent frame. We further postdilated the stent after valve deployment to maintain the natural horizontal axis for future coronary engagement.

The final angiogram demonstrated excellent results with patency of the RCA with no visible distortion of the coronary stent (Figure 10). There was no paravalvular leak seen on angiogram or on transthoracic echocardiogram. Given placement of the RCA stent 4 ​weeks prior to patients’ TAVR, dual antiplatelet therapy was continued during and after the procedure. The duration of antiplatelet therapy is to be continued according to standard guidelines.

## Discussion

This report describes a challenging scenario TAVR operators face in dealing with protruding ostial coronary stents. Optimal deployment of coronary stents in the aorto-ostial segment remains a challenge. Procedural and anatomical considerations including calcification, coronary angulation, and appropriate visualization of the true ostium must be considered during stent placement. The optimal fluoroscopic angle that has been described based on computed tomographic coronary angiography for ostial right coronary stenting was left anterior oblique 79°, cranial 41°.^2^ In addition, various interventional procedural techniques have been described.^3^

Protruding ostial stents can present many challenges. Cases of acute perforation of the coronary cusp and severe aortic insufficiency have been described. Protruding stents into the aorta are at a higher risk of strain, putting them at increased risk for embolization and stent fracture.^4^

Given the rapidly evolving structural field, consideration of the type of aortic valve must be considered. As described above, one should consider simultaneous kissing balloon inflation to prevent stent distortion and compromise of coronary flow. Choosing a balloon-expandable valve over a self-expanding one was important, as it would be very difficult to maintain and preserve the coronary stent architecture after the release of a self-expandable valve. This case highlights the importance of planning a TAVR procedure and thinking about the whole aortic valve complex including ostial coronary stents.

## Consent Statement

Consent was obtained from the patient for publishing this article and accompanying images.

## Funding

Funding for publishing this article was provided by the Baylor Research Institute.

## Disclosure statement

Karim Al-Azizi is a consultant and proctor for Edwards LifeSciences and is a member of the advisory board for Medtronic. The other authors had no conflicts to declare.
